# Evaluating a pre-surgical health optimisation programme: a feasibility study

**DOI:** 10.1186/s13741-022-00255-2

**Published:** 2022-06-23

**Authors:** Joanna McLaughlin, Lauren J. Scott, Lucie Owens, Hugh McLeod, Carlos Sillero-Rejon, Rebecca Reynolds, Amanda Owen-Smith, Elizabeth M. Hill, Russell Jago, Jenny L. Donovan, Sabi Redwood, Ruth Kipping

**Affiliations:** 1 Population Health Sciences, Bristol Medical School, University of Bristol, Bristol, UK; 2 National Institute for Health Research Applied Research Collaboration West (NIHR ARC West), University Hospitals Bristol and Weston NHS Foundation Trust, Bristol, UK; 3 NHS Bath and North East Somerset, Swindon and Wiltshire Clinical Commissioning Groups, Chippenham, UK; 4 Bath and North East Somerset Council, Bath, UK; 5 Centre for Exercise Nutrition & Health Sciences, School for Policy Studies, University of Bristol, Bristol, UK

**Keywords:** Health optimisation, Prehabilitation, Obesity, Smoking, Elective surgery

## Abstract

**Background:**

Health optimisation programmes are increasingly popular and aim to support patients to lose weight or stop smoking ahead of surgery, yet there is little published evidence about their impact. This study aimed to assess the feasibility of evaluating a programme introduced by a National Health Service (NHS) clinical commissioning group offering support to smokers/obese patients in an extra 3 months prior to the elective hip/knee surgery pathway.

**Methods:**

Feasibility study mapping routinely collected data sources, availability and completeness for 502 patients referred to the hip/knee pathway in February–July 2018.

**Results:**

Data collation across seven sources was complex. Data completeness for smoking and ethnicity was poor. While 37% (184) of patients were eligible for health optimisation, only 28% of this comparatively deprived patient group accepted referral to the support offered. Patients who accepted referral to support and completed the programme had a larger median reduction in BMI than those who did not accept referral (− 1.8 BMI points vs. − 0.5). Forty-nine per cent of patients who accepted support were subsequently referred to surgery, compared to 61% who did not accept referral to support.

**Conclusions:**

Use of routinely collected data to evaluate health optimisation programmes is feasible though demanding. Indications of the positive effects of health optimisation interventions from this study and existing literature suggest that the challenge of programme evaluation should be prioritised; longer-term evaluation of costs and outcomes is warranted to inform health optimisation policy development.

**Supplementary Information:**

The online version contains supplementary material available at 10.1186/s13741-022-00255-2.

## Background

Pathways to surgery are being redesigned with the increased use of ‘health optimisation’ or ‘prehabilitation’ programmes across the NHS and internationally (Grocott et al., 2017). Their purpose is often to encourage eligible patients to lose weight, stop smoking and increase fitness ahead of surgery. The intended outcomes range from reduction in surgical procedures, improved outcomes and recovery from surgery, and taking the wider public health opportunity offered by consideration of surgery to trigger lasting lifestyle change (Durrand et al., 2019; NHS South West Clinical Senate, 2017). In England, Clinical Commissioning Groups (CCGs) are clinically-led statutory NHS bodies responsible for the planning and commissioning of healthcare services for their local area. It is estimated that over a third of CCGs have introduced such a programme, in part to manage demand for surgery and reduce costs (Royal College of Surgeons of England, 2016). In 2015, 83% of CCGs restricted access to some healthcare based on body mass index (BMI) and 62% based on smoking status (Millett, 2015). There are currently no NICE guidelines on the content and nature of such programmes, and much variation in the programmes that have been introduced. Despite many programmes being in operation for several years, few evaluations have been published and evidence for their effectiveness and potential unintended consequences remains uncertain (Durrand et al., 2019; Pillutla et al., 2018).

**Fig. 1:**
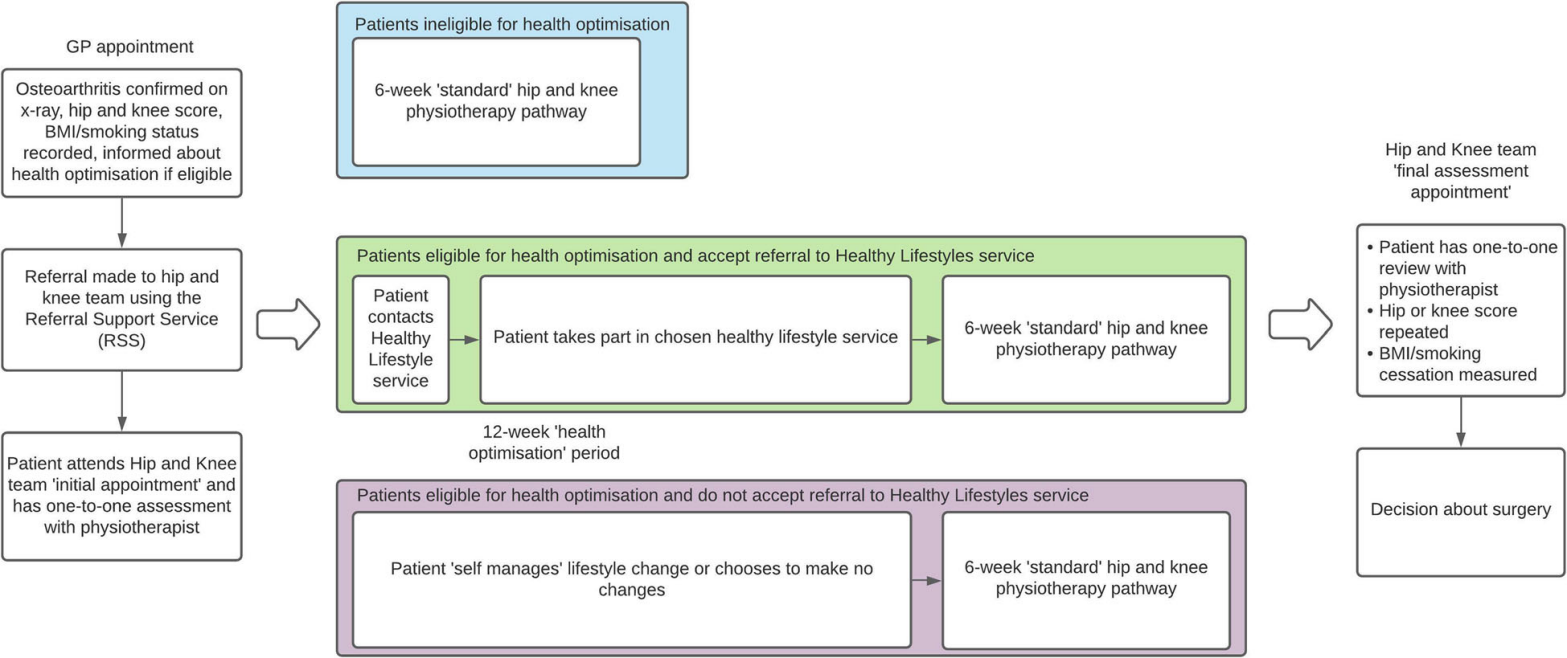
The hip and knee pathway for health optimisation and non-health optimisation patients. Note: Non-health optimisation patients proceed immediately from the initial Hip and Knee team appointment to the 6-week ‘standard’ hip and knee pathway

In 2017, a CCG in South West England introduced a health optimisation policy whereby patients who are obese (BMI ≥ 30 kg/m^2^) or smoke were offered support to lose weight or stop smoking in a 3-month period prior to referral for elective hip or knee surgery. The health optimisation programme consisted of referral to the existing ‘Healthy Lifestyle Services’ commissioned by local authority public health and provided by Virgin Care) where patients could select from options including slimming group vouchers, one-to-one weight management service (‘Counterweight’ (Counterweight Project T, 2008)) and individual face-to-face smoking cessation support programmes, for the 3-month period. Referrals were made by the physiotherapy-led Hip and Knee team who receive all hip and knee referrals from general practice via the Referral Support Service (see Fig. 1). All eligible patients were required to wait 3 months whether or not they accepted support, after which they completed the existing ‘hip and knee pathway’—a 6-week physiotherapy programme culminating in a referral for surgery should surgery be indicated. There was no penalty for those who were unsuccessful in weight loss or stopping smoking, or those who chose not to engage with the Healthy Lifestyles Service. The CCG undertook a 10-week period of public consultation prior to introducing the programme which indicated support for the policy (Bath and North East Somerset Clinical Commissioning Group, 2018).

The aim of this study was to:

- Map pathways for hip and knee patients eligible or ineligible for health optimisation through the various health services from referral to discharge
- Evaluate the feasibility of accessing and collating routinely collected data on patient characteristics and progression through the pathways, for the purpose of evaluation
- Draw insight from the descriptive summaries of these data.

The experiences of implementing the new policy from the perspectives of clinicians, commissioners, and patients were also investigated and have been published separately (McLaughlin et al., 2021).

## Methods

### Patients and setting

The study population were patients in the CCG region referred into the NHS hip and knee pathway from general practice between February and July 2018 inclusive; patients were followed up for 12 months. Patients were categorised on whether they followed the health optimisation pathway or the standard (non-health optimisation) pathway (see Fig. 1). Health optimisation patients were compared to non-health optimisation patients, and comparisons were also made between health optimisation patients who did and did not accept referral into the Healthy Lifestyles Service.

### Data collection

The services involved in the standard hip and knee pathway included general practice, the Referral Support Service, Virgin Care’s hip and knee physiotherapy team, and in-hospital medical and surgical teams for patients referred for surgery. In addition, health optimisation patients could also interact with weight management and smoking cessation services via the Healthy Lifestyles Service (see Fig. 1). All relevant available routine data from the services were collated centrally by the local Commissioning Support Unit and pseudo-anonymised before secure transfer to the research team for analysis. Additional file 1 provides details of the data sources. Data included demographics, hip and knee clinical measures including Oxford score (Dawson et al., 1996) and surgical hospital episodes, BMI and smoking status, and Healthy Lifestyles Service referral. The Oxford score is an outcome measurement tool designed to assess disability in patients under consideration for joint replacement surgery. Scoring involves summing totals for 12 questionnaire items to produce a final score between 0 and 48, with higher scores indicating less disability.

### Analysis

Quantitative data were summarised using means and standard deviations (SD), medians and interquartile ranges (IQR) or counts and percentages, as appropriate. As this was a feasibility study, no formal statistical comparisons were made. BMI, smoking status, and Oxford score were measured at GP referral and again at physiotherapy discharge; changes in BMI and Oxford score were calculated as physiotherapy discharge score minus GP referral score. Length of time for movements through services (e.g. time from initial physiotherapy assessment to follow-up) was calculated as the date of the second time point of interest minus the date of the first (for example physiotherapy follow-up date minus initial physiotherapy assessment date). All quantitative data management and analysis were performed using Stata 15.1.

Costs for the pathway from initial GP appointment, referral, hip and knee physiotherapy team, surgery and rehabilitation were estimated from a health system perspective using national routine data sources (Curtis & Burns, 2018; NHS Improvement, 2018). The additional costs for health optimisation patients who interacted with smoking cessation and weight management services were estimated through national routine data sources (NHS Digital, 2018) or relevant literature (Ahern et al., 2017; Better: The feel good place, 2021; Slimming world, 2021; Trueman et al., 2010). Costs are expressed in 2017/2018 prices.

### Patient and public involvement

The National Institute for Health Research Applied Research Collaboration (ARC) West’s Patient and Public Involvement group were engaged during the study design, to understand their thoughts about the health optimisation policy and where they felt the research should be focused. Their contribution encouraged further consideration of the outcomes and experiences of vulnerable groups.

## Results

### Feasibility of data collection

Mapping the patient pathway from initial general practice referral to discharge identified seven services with potential patient contact and therefore sources of data pertinent to the evaluation; primary care, the Referral Support Service, physiotherapy team, Healthy Lifestyle Service, community-based smoking cessation services, community-based weight management services and secondary care at the acute trust (see Additional file 1). General practice data were only indirectly available by accessing the information provided to the Referral Support Service. This provided the main source of demographic data. Data from each source were not routinely integrated, for example, general practice did not receive information on the patient’s interaction with the Healthy Lifestyles Service. Information technology systems within the organisations did not allow automatic data collation, as many data sources consisted of manually populated spreadsheets. It took 12 months to agree on the data collation, information governance arrangements and provision to the research team. Honorary NHS Clinical Support Unit contracts for the research team were instrumental in allowing data provision.

### Data completeness

Age and sex were available for all patients. Ethnicity was poorly recorded in general practice and was often mixed up with nationality, so is not reported here. Baseline BMI and Oxford scores were well completed (96% and 99%, respectively), and for patients who completed the referral pathway, Oxford scores were also well recorded at the end of the pathway (95%). In contrast, BMI was poorly recorded at the end of the pathway for these patients (52%), though was better recorded for health optimisation patients than non-health optimisation patients (68% and 45%, respectively). Smoking at baseline appeared to be under-reported compared to population figures (5% in our study compared to 11.7% of adults in the CCG region in 2018 (Public Health England, 2019)) and was not collected at follow-up for any patients who completed the pathway, so changes to smoking status could not be assessed.

### Patient population and baseline characteristics

During the 6-month study period, 502 patients were referred to the NHS elective hip/knee surgery pathway from general practice. Of these, 37% (184) were referred for health optimisation: 91% (168) were obese, 11% (29) were smokers, 7% (13) were both and 5% (9) did not appear to meet either criterion.

The health optimisation group compared to the non-health optimisation group were similar in age and sex but were more socioeconomically deprived (Table 1). The health optimisation group also included a larger proportion of knee patients (compared to hip patients) than the non-health optimisation group (73.4% vs. 58.2%). Patients had worse average baseline Oxford scores in the health optimisation group than the non-health optimisation group (median 16 vs. 19). As expected, there were more smokers in the health optimisation group than the non-health optimisation group (11% vs. 2%), and the health optimisation group had a higher median BMI (33 vs. 26). Within the health optimisation group, patients who accepted referral to the Healthy Lifestyles Service were more likely to be female and deprived than those who did not accept referral. They also had slightly higher average BMI, were more likely to smoke and had worse Oxford scores (Table 1).

**Table 1:** Baseline information by patient group (health optimisation and non-health optimisation)

	Non-health optimisation pathway		Health optimisation pathway					
			Accepted referral to Healthy Lifestyles Service		Did not accept referral to Healthy Lifestyles Service		All health optimisation patients (accepted referral & did not accept referral combined)	
	(***n***=318)		(***n***=51)		(***n***=133)		(***n***=184)	
**Sex:**								
Female	195	61.3%	36	70.6%	79	59.4%	115	62.5%
Male	123	38.7%	15	29.4%	54	40.6%	69	37.5%
**Age (years):**								
Median, IQR	72	(66, 79)	67	(58, 74)	70	(63, 75)	70	(62, 75)
**Deprivation quintile:**								
1 (most deprived)	10	3.1%	3	5.9%	11	8.3%	14	7.6%
2	23	7.2%	10	19.6%	8	6.0%	18	9.8%
3	34	10.7%	8	15.7%	29	21.8%	37	20.1%
4	120	37.7%	20	39.2%	42	31.6%	62	33.7%
5 (least deprived)	131	41.2%	10	19.6%	43	32.3%	53	28.8%
**Referral reason:**								
Hip	133	41.8%	15	29.4%	34	25.6%	49	26.6%
Knee	185	58.2%	36	70.6%	99	74.4%	135	73.4%
**Smoking status:**								
Smoker	5	1.6%	8	15.7%	12	9.0%	20	10.9%
Non-smoker	297	93.4%	41	80.4%	118	88.7%	159	86.4%
Missing	16	5.0%	2	3.9%	3	2.3%	5	2.7%
**BMI (kg/m**^**2**^**):**								
< 18: underweight	1	0.3%	0	0.0%	0	0.0%	0	0.0%
18–24: healthy weight	99	31.1%	1	2.0%	3	2.3%	4	2.2%
25–29: overweight	192	60.4%	0	0.0%	11	8.3%	11	6.0%
30+: obese	9	2.8%	50	98.0%	117	88.0%	167	90.8%
Missing	17	5.3%	0	0.0%	2	1.5%	2	1.1%
Median, IQR ^a^	26.0	(24.0, 28.0)	35.0	(32.0, 38.2)	33.0	(31.0, 35.6)	33.2	(31.4, 36.3)
**Oxford score:**								
0–19: severe	158	49.7%	37	72.5%	80	60.2%	117	63.6%
20–29: moderate	149	46.9%	13	25.5%	50	37.6%	63	34.2%
30–39: mild	6	1.9%	0	0.0%	2	1.5%	2	1.1%
40–48: normal	0	0.0%	0	0.0%	1	0.8%	1	0.5%
Missing	5	1.6%	1	2.0%	0	0.0%	1	0.5%
Median, IQR *	19	(15, 24)	14	(11, 20)	17	(12, 22)	16	(12, 21)

*BMI* body mass index, *IQR* interquartile range

^a^For those patients without missing data

### Movement through pathway

Patient engagement with the hip and knee pathway was variable. Only 68% of patients in the health optimisation group and 78% of other patients completed the pathway (Table 2). In part, this was because fewer patients in the health optimisation group attended the 6-week physiotherapy component (76% vs. 81%) that was viewed as forming an integral part of the pathway for all patients (see Fig. 1), and more patients in the health optimisation group dropped out of the pathway (8% vs. 3%) (Table 2). In addition, fewer patients in the health optimisation group were referred to self-management compared to other patients (26% vs. 32%) (Table 2).

**Table 2:** Patient outcomes by group for all patients referred into the Hip and Knee pathway: surgery or self-management

	Non-health optimisation pathway^a^		Health optimisation pathway^a^					
			Accepted referral to Healthy Lifestyles Service		Did not accept referral to Healthy Lifestyles Service		All health optimisation patients (accepted referral & did not accept referral combined)	
	(***n***=318)		(***n***=51)		(***n***=133)		(***n***=184)	
**Referred for surgery**	179	56.3%	25	49.0%	81	60.9%	106	57.6%
Completed physiotherapy	155	86.6%					88	83.0%
**Referred for self-management**	103	32.4%	17	33.3%	31	23.3%	48	26.1%
Completed physiotherapy	94	91.3%					37	77.1%
**No onward referral:**	36	11.3%	9	17.6%	21	15.8%	30	16.3%
Never joined the pathway	22	6.9%					13	7.1%
Dropped out of pathway	9	2.8%					14	7.6%
Still in the pathway	4	1.3%					2	1.1%
Died	1	0.3%					1	0.5%
Duration of physiotherapy (months)^b^, median (IQR)	1.8	(1.5, 2.3)	4.1	(3.8, 4.4)	3.7	(1.8, 4.1)	3.9	(2.1, 4.2)
Length of hospital stay (days)^c^, median (IQR)	3	(2, 3)					3	(2,4)

^a^249/318 (78.3%) on the non-health optimisation and 125/184 (67.9%) on the health optimisation ‘completed’ the pathways (attended the core physiotherapy element and a final assessment appointment)

^b^From initial assessment to follow-up appointment for patients who completed physiotherapy

^c^For patients who went on to be admitted for surgery during the follow-up period

Only 28% (51/184) of the health optimisation group accepted referral to the Healthy Lifestyles Service. Nearly all (49/51) of these referrals were to weight management services (such as Weightwatchers/Slimming World) and 4% (2/51) were to smoking cessation services. While the proportion of patients referred for surgery was similar for health optimisation group patients and other patients, fewer health optimisation group patients who accepted referral to the Healthy Lifestyles Service were referred for surgery (49%) compared to those (61%) who did not (Table 2). The comparatively high proportion of health optimisation group patients who did not accept a referral for support and were referred to surgery was matched by a comparatively low proportion of these patients referred to self-management (23%) compared to those referred to the Healthy Lifestyles Service (33%) or other patients (32%) (Table 2). Table 2 records the reasons for no onward referral.

For patients who completed physiotherapy, the duration of physiotherapy treatment was longer for health optimisation patients compared to non-health optimisation patients, and there is no difference in length of stay for these patients who went on to be admitted for surgery during the follow-up period (Table 2).

### Outcomes

Repeat measures of BMI, smoking status and Oxford score were not recorded unless patients completed physiotherapy and attended a final assessment appointment. For these patients, 31% (39/125) of the health optimisation group reduced their BMI by at least 2 kg/m^2^ compared to 4% (10/249) of the non-health optimisation group. Changes in median BMI are shown in Table 3. Thirteen per cent (16/125) of the health optimisation group and 24% (60/249) of the non-health optimisation group moved from ‘severe/moderate’ to ‘mild/normal’ Oxford score categories. Health optimisation patients who accepted referral to the Healthy Lifestyles Service had a larger median reduction in BMI than those who did not accept referral (− 1.8 vs. − 0.5) (Table 3). There was no difference between these groups in median change in Oxford score (Table 3).

**Table 3:** Patient outcomes by group for those who completed the physiotherapy pathway: BMI and Oxford scores

	Non-health optimisation pathway				Health optimisation pathway											
					Accepted referral to Healthy Lifestyles Service				Did not accept referral to Healthy Lifestyles Service				All health optimisation patients (accepted referral & did not accept referral combined)			
	***n***=249				***n***=34				***n***=91				***n***=125			
	Baseline		Follow-up		Baseline		Follow-up		Baseline		Follow-up		Baseline		Follow-up	
**BMI (kg/m**^**2**^**):**																
< 18: underweight	0	0.0%	0	0.0%	0	0.0%	0	0.0%	0	0.0%	0	0.0%	0	0.0%	0	0.0%
18–24: healthy weight	72	28.9%	34	13.7%	0	0.0%	0	0.0%	0	0.0%	0	0.0%	0	0.0%	0	0.0%
25–29: overweight	163	65.5%	72	28.9%	0	0.0%	3	8.8%	8	8.8%	13	14.3%	8	6.4%	16	12.8%
30+: obese	6	2.4%	5	2.0%	34	100.0%	20	58.8%	81	89.0%	49	53.8%	115	92.0%	69	55.2%
Missing	8	3.2%	138	55.4%	0	0.0%	11	32.4%	2	2.2%	29	31.9%	2	1.6%	40	32.0%
Median, IQR*	26.0	(24.0, 28.0)	26.0	(24.0, 28.4)	34.8	(32.0, 38.2)	34.0	(31.0, 38.0)	33.0	(31.0, 36.3)	32.0	(30.0, 35.6)	33.2	(31.4, 37.0)	32.1	(30.0, 36.4)
Change in BMI			0.0	(0.0, 0.0)			− 1.8	(− 3.9, − 0.5)			− 0.5	(− 1.8, 0.0)			− 0.8	(− 2.4, 0.0)
**Oxford score:**																
0–19: severe	123	49.4%	87	34.9%	23	67.60%	15	44.1%	56	61.5%	44	48.4%	79	63.2%	59	47.2%
20–29: moderate	122	49.0%	88	35.3%	11	32.4%	13	38.2%	34	37.4%	31	34.1%	45	36.0%	44	35.2%
30–39: mild	4	1.6%	51	20.5%	0	0.0%	3	8.8%	1	1.1%	12	13.2%	1	0.8%	15	12.0%
40–48: normal	0	0.0%	10	4.0%	0	0.0%	0	0.0%	0	0.0%	1	1.1%	0	0.0%	1	0.8%
Missing	0	0.0%	13	5.2%	0	0.0%	3	8.8%	0	0.0%	3	3.3%	0	0.0%	6	4.8%
Median, IQR*	20	(15, 25)	23	(17, 30)	16	(11, 21)	21	(13, 25)	18	(13, 22)	20	(13, 25)	17	(12, 21)	20	(13, 25)
Change in Oxford score			3	(− 1, 8)			1	(− 3, 8)			1	(− 2, 7)			1	(− 2, 7)

*BMI* body mass index, *IQR* interquartile range

^a^For those patients without missing data

#### Costings

National reference cost data indicate that the mean cost for the standard surgery pathway, from initial general practitioner (GP) appointment to rehabilitation, was £6883 for knee patients and £7110 for hip patients (see Additional file 2). This included the estimated cost of £105 for the physiotherapy-led element of care (hip and knee team) for patients who fully engaged with it. For health optimisation patients who accepted a weight management referral, literature-based data suggested a mean cost of £57 (across a range of £40 to £75), and local NHS smoking cessation support cost of £505 per participant (see Additional file 2).

## Discussion

### Main findings of this study

Collating routine data from seven sources to evaluate a health optimisation programme was difficult, but ultimately possible. Some important data were unavailable or missing and many patients did not progress through the pathway as expected (for example, 21% never attended a physiotherapy appointment).

Over a third (37%, 184/502) of patients referred into the hip and knee pathway were eligible for health optimisation; however, less than a third of this comparatively deprived patient group (28%) accepted referral to the Healthy Lifestyles Service.

In the health optimisation group, 49% of patients who accepted referral to the Healthy Lifestyles Service were referred to surgery, compared to 61% who did not accept referral, and 56% in the non-health optimisation group. On average all patients in all groups experienced improvement in Oxford scores, particularly in terms of the reduction in the proportion with ‘severe’ scores at follow-up. However, the health optimisation group patients experienced comparatively less improvement to ‘mild’ or ‘normal’ scores compared to those in the non-health optimisation group. This finding suggests that the need for surgery remained comparatively high for those in the health optimisation group compared to the other patients.

Health optimisation patients reduced their BMI more than non-health optimisation patients; this was even greater for patients who accepted referral to the Healthy Lifestyles Service. The estimated additional intervention cost to support a health optimisation patient was approximately 1% (£57) of the standard surgical pathway for weight management, rising to about 7% (£505) for smoking cessation.

### What is already known on this topic

Few published papers report on pre-surgical health optimisation programmes and none have used routine data alone—instead, more resource-intensive patient recruitment such as in the PREP-WELL programme (Danjoux et al., 2019), or full randomised controlled trials, are used to determine the impact of interventions (Liljensoe et al., 2019; Lindstrom et al., 2008; Villebro et al., 2008). Evaluation of healthcare interventions using routine data is supported by recent guidance in the British Medical Journal (Clarke et al., 2019) though the authors noted that, similar to our experience—as routine datasets are designed to support direct care and administrative purposes rather than research, ‘the use of routinely collected data for evaluating changes in health service delivery is not without pitfalls’. They also note that information governance arrangements and a central pseudonymisation process are crucial elements. Further, Franklin and Thorn (2019) reported that routine electronic data may be more accurate and more practical than use of patient-reported resource use (Franklin & Thorn, 2019), supporting our decision to use routine data.

A recent systematic review of trials of pre-admission interventions to improve elective surgery outcomes (Perry et al., 2021) found only low-quality evidence from three studies in the setting of orthopaedic surgery. However, there are other non-randomised trials in the UK and America which report positive impact of health optimisation (Clarke et al., 2019; Bernstein et al., 2018).

### Limitations of this study

Most limitations pertain to gaps in data collection and completeness, which is likely to be a function of working across multiple providers and data systems. The 12-month follow-up period did not capture all proposed surgeries; approximately half were still on the waiting list. Data on uptake of the Healthy Lifestyles Service support offers were not routinely collected so could not be evaluated. Similarly, self-management of weight or smoking, or referral to external sources of support were not captured. General practice data were obtained through the Referral Support Service, so data on comorbidities, disabilities, and general practice use during wait for surgery were not available. Further, baseline smoking rates were lower than expected; the referral method relied on self-report which may have led to under-reporting. This, and the lack of follow-up data on smoking status, meant it was not possible to see the impact of the programme on smoking cessation. However, exploring data availability was a key part of this feasibility study, so all these data issues are important study findings which will help guide future evaluations. Developments in the quality of information technology systems, and the integration of these systems between services over time, will likely result in improvements in data completeness and accessibility in many settings.

## Conclusions

We believe this is the first study to report on the practicalities of using routine data to evaluate a surgical health optimisation referral pathway, and therefore should provide a route map for planning future programme evaluations. Policy makers should address data availability before new policies are introduced to allow determination of the programme impact. Provision should be made for long follow-up periods, including economic measures, to assess whether there are public health benefits from sustained healthy weight or smoking cessation. If, as here, health optimisation programmes disproportionately involve deprived populations, future research should investigate whether health optimisation may tackle health inequality by offering an effective way to engage particular groups with health improvement.

This study’s data suggest that on average obese patients who do engage with support services reduce their BMI and may be less likely to be referred for surgery than those who do not. If this is due to symptom improvement such that surgery is no longer needed, then NHS resources will be saved. However, further investigation is required to understand if this is the case rather than due to other reasons, for example, unwarranted referral of some patients to self-management or to some vulnerable patients dropping out from services. Furthermore, it may be that the health optimisation intervention supports some patients to reduce their BMI such that they are referred for surgery when this would not have been viewed as appropriate with their higher baseline BMI, and here the benefits are likely to outweigh the costs. The low levels of engagement with support services reflect the well-known barriers to uptake of weight management or smoking cessation interventions (McVay et al., 2018; Public Health England, 2018). Expansion of routine data collection to capture reasons for low engagement and drop out would be of benefit. Our related qualitative study reports on patient, clinician and commissioner insight into the experience of using a health optimisation programme to guide recommendations for programme modifications (McLaughlin et al., 2021).

The positive indications of health optimisation interventions’ effects on BMI and clinical scores from this study and existing literature indicate that the challenge of evaluation of the policies in the UK system should be taken on as a priority. More extensive and experimental research designs would facilitate additional insight on the impact of health optimisation interventions.

## Supplementary Information

**Additional file 1.** Flow chart of data sources

**Additional file 2.** Costings; Estimated hip and knee pathway-related component costs

## Data Availability

The datasets used and/or analysed during the current study are available from the corresponding author on reasonable request.

## Abbreviations

ARC: Applied Research Collaboration
BMI: Body mass index
CCG: Clinical commissioning group
GP: General practitioner
HO: Health optimisation
IQR: Inter quartile range
SD: Standard deviation

## References

[CR1] Ahern AL, Wheeler GM, Aveyard P, Boyland EJ, Halford JCG, Mander AP, et al. Extended and standard duration weight-loss programme referrals for adults in primary care (WRAP): a randomised controlled trial. Lancet. 2017;389(10085):2214–25. 10.1016/S0140-6736(17)30647-5. 10.1016/S0140-6736(17)30647-5 PMC5459752 28478041

[CR2] Bath and North East Somerset Clinical Commissioning Group (2018). Our Consultation in Numbers; Public feedback on our plans to support patients to try to stop smoking and lose weight before they are referred for non-urgent operations.

[CR3] Bernstein DN, Liu TC, Winegar AL, Jackson LW, Darnutzer JL, Wulf KM, et al. Evaluation of a Preoperative Optimization Protocol for Primary Hip and Knee Arthroplasty Patients. J Arthroplast. 2018;33(12):3642–8. 10.1016/j.arth.2018.08.018. 10.1016/j.arth.2018.08.018 30201213

[CR4] Better: The feel good place. https://www.better.org.uk/. Accessed 1 Sept 2021.

[CR5] Clarke GM, Conti S, Wolters AT, Steventon A (2019). Evaluating the impact of healthcare interventions using routine data. BMJ..

[CR6] Counterweight Project T (2008). Evaluation of the Counterweight Programme for obesity management in primary care: a starting point for continuous improvement. Br J Gen Pract.

[CR7] Curtis L, Burns A (2018). Unit Costs of Health and Social Care 2018.

[CR8] Danjoux G, Tew G, Carr E, Gray J (2019). Preparing For Surgery: The Community Prehabilitation and Wellbeing Project (The PREP-WELL Project).

[CR9] Dawson J, Fitzpatrick R, Carr A, Murray D (1996). Questionnaire on the perceptions of patients about total hip replacement. J Bone Joint Surg (Br).

[CR10] Durrand J, Singh SJ, Danjoux G (2019). Prehabilitation. Clin Med (Lond).

[CR11] Franklin M, Thorn J (2019). Self-reported and routinely collected electronic healthcare resource-use data for trial-based economic evaluations: the current state of play in England and considerations for the future. BMC Med Res Methodol.

[CR12] Grocott MPW, Plumb JOM, Edwards M, Fecher-Jones I, Levett DZH (2017). Re-designing the pathway to surgery: better care and added value. Perioper Med (Lond).

[CR13] Liljensoe A, Laursen JO, Bliddal H, Soballe K, Mechlenburg I (2019). Weight Loss Intervention Before Total Knee Replacement: A 12-Month Randomized Controlled Trial. Scand J Surg.

[CR14] Lindstrom D, Sadr Azodi O, Wladis A, Tonnesen H, Linder S, Nasell H (2008). Effects of a perioperative smoking cessation intervention on postoperative complications: a randomized trial. Ann Surg.

[CR15] McLaughlin J, Palmer C, Redwood S, Kipping R, Owens L, Reynolds R, et al. Commissioner, clinician, and patient experiences of a pre-surgical health optimisation programme – a qualitative study. BMC Health Serv Res. 2021;21(1):409. 10.1186/s12913-021-06434-z. 10.1186/s12913-021-06434-z PMC8088197 33933095

[CR16] McVay MA, Yancy WS, Bennett GG, Jung SH, Voils CI (2018). Perceived barriers and facilitators of initiation of behavioral weight loss interventions among adults with obesity: a qualitative study. BMC Public Health.

[CR17] Millett D (2015). Exclusive: NHS care rationed for smokers and obese. GP online.

[CR18] NHS Digital (2018). Statistics on NHS Stop Smoking Services in England - April 2017 to March 2018.

[CR19] NHS Improvement (2018). National Cost Collection for the NHS: 2017/18 reference costs.

[CR20] NHS South West Clinical Senate (2017). Clinical Senate Council Meeting.

[CR21] Perry R, Herbert G, Atkinson C, England C, Northstone K, Baos S, Brush T, Chong A, Ness A, Harris J, Haase A, Shah S, Pufulete M. Pre-admission interventions (prehabilitation) to improve outcome after major elective surgery: a systematic review and meta-analysis. BMJ Open. 2021 Sep 30;11(9):e050806. 10.1136/bmjopen-2021-050806. 10.1136/bmjopen-2021-050806 PMC8487197 34593498

[CR22] Pillutla V, Maslen H, Savulescu J (2018). Rationing elective surgery for smokers and obese patients: responsibility or prognosis?. BMC Med Ethics.

[CR23] Public Health England (2018). Uptake and retention in group-based weight-management services: Literature review and behavioural analysis.

[CR24] Public Health England (2019). Local Tobacco Control Profiles.

[CR25] Royal College of Surgeons of England (2016). Smokers and Overweight Patients: Soft Targets for NHS savings?.

[CR26] Slimming world. How much does it cost? https://www.slimmingworld.co.uk/health/swor/how-much-does-it-cost.aspx. Accessed 1 Sept 2021.

[CR27] Trueman P, Haynes SM, Felicity Lyons G, Louise McCombie E, McQuigg MS, Mongia S, et al. Long-term cost-effectiveness of weight management in primary care. Int J Clin Pract. 2010;64(6):775–83. 10.1111/j.1742-1241.2010.02349.x. 10.1111/j.1742-1241.2010.02349.x 20353431

[CR28] Villebro NM, Pedersen T, Moller AM, Tonnesen H (2008). Long-term effects of a preoperative smoking cessation programme. Clin Respir J.

