# Supplementary figures and images for: Evaluating a pre-surgical health optimisation programme: a feasibility study
Source: Perioper Med (Lond). 2022 Jun 23;11:21. doi: 10.1186/s13741-022-00255-2 (PMC9219203; doi:10.1186/s13741-022-00255-2)

**Additional file 1 - Flow chart of data sources**


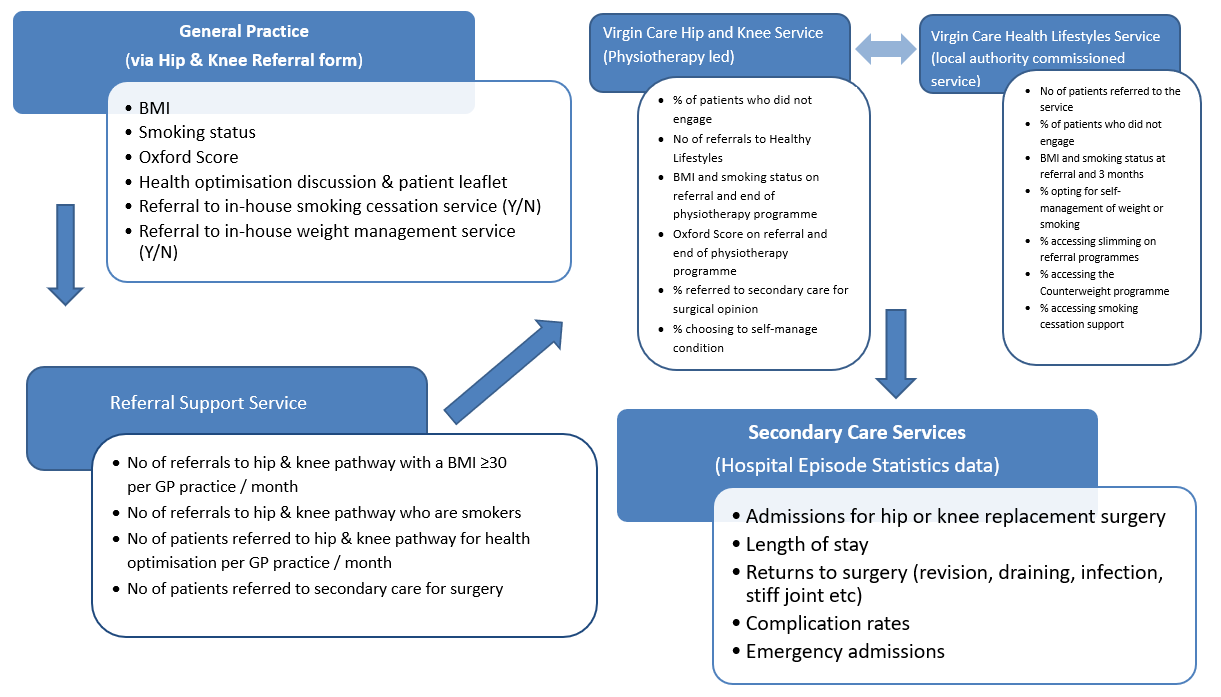

Supplement: Supplementary file 1 — Additional file 1. Flow chart of data sources [file 13741_2022_255_MOESM1_ESM.docx]
