# Supplementary material for: Evaluating a pre-surgical health optimisation programme: a feasibility study
Source: Perioper Med (Lond). 2022 Jun 23;11:21. doi: 10.1186/s13741-022-00255-2 (PMC9219203; doi:10.1186/s13741-022-00255-2)
Supplement: Supplementary file 2 — Additional file 2. Costings; Estimated hip and knee pathway-related component costs [file 13741_2022_255_MOESM2_ESM.docx]

**Additional file 2 - Costings**

**Estimated hip and knee pathway-related component costs**

| Component | Cost £ (2017 prices) | Sources |
| --- | --- | --- |
| Health Optimisation programme  Healthy Lifestyle Services^1^  Weight management interventions^2^ |  |  |
| Counterweight | 75.11 | [12] |
| Weight Watchers | 62.86 | [25] |
| Slimming World | 49.50 | [26] |
| Healthwise | 39.60 | [27] |
| NHS Stop Smoking Services^3^ | 505.26 | [11] |
| Standard surgical pathway |  |  |
| Referral support service^4^  Hip and Knee team pathway^5^  General Practitioner appointment | 6.00  105.19  37.00 | [9]  [9]  [9] |
| Direct Access Plain Film (X-Ray)^6^ | 31.00 | [10] |
| Orthopaedic consultant-led outpatient appointment | 128.00 | [10] |
| Knee replacement surgery^7^ | 6,251.00 | [10] |
| Hip replacement surgery^8^ | 6,478.00 | [10] |
| Rehabilitation^9^ | 325.00 | [10] |

^1^ In the absence of local Healthy Lifestyle Services cost data, these costs are sourced from literature and routine data on local NHS Stop Smoking Services.
^2^ The mean cost of these weight management interventions is £56.77. ^3^ BANES Local Authority spent £309,728 on smoking cessation including pharmacotherapies in 2017/18 and 613 people enrolled and set a quit date, and it is assumed that the cost of the Healthy Lifestyle Service cession intervention is similar.
^4^ Assumes the cost is similar to that for nurse-led triage in primary care.
^5^ The estimated cost is based on a 120 minute initial group assessment staffed by 1 Band 4 exercise instructor, 1 Band 5 physiotherapist and 1 Band 6 physiotherapist, a 60 minute final assessment appointment with 1 band 6 physiotherapist, and six weekly 90 minute physiotherapy group sessions each with 1 band 4 physiotherapist, which includes one 30 minute lifestyle management session provided by 1 band 4 member of the Health Improvement Team. It is assumed that on average eight patients attend each group session. The band 6, 5 and 4 unit costs per hour are £44, £34 and £29, respectively.
^6^ Healthcare Resource Group (HRG) DAPF
^7^ National mean weighted cost for HRGs HN22A, HN22B, HN22C, HN22D, HN22E.
^8^ National mean weighted cost for HRGs HN12A, HN12B, HN12C, HN12D, HN12E, HN12F.
^9^ For joint replacement, non-specialist rehabilitation services level 3 (VC18Z).
